# Novel Mutation in *CRYBB3* Causing Pediatric Cataract and Microphthalmia

**DOI:** 10.3390/genes12071069

**Published:** 2021-07-13

**Authors:** Olivia A. Zin, Luiza M. Neves, Fabiana L. Motta, Dafne D. G. Horovitz, Leticia Guida, Leonardo H. F. Gomes, Daniela P. Cunha, Ana Paula S. Rodrigues, Andrea A. Zin, Juliana M. F. Sallum, Zilton F. M. Vasconcelos

**Affiliations:** 1 Department of Ophthalmology, Universidade Federal de São Paulo, Sao Paulo 04039-032, Brazil; olivia.zin@gmail.com (O.A.Z.); anapaulasilveriorodrigues@gmail.com (A.P.S.R.); juliana@pobox.com (J.M.F.S.); 2 Instituto Fernandes Figueira-Fundação Oswaldo Cruz, Rio de Janeiro 22250-020, Brazil; luiza.macneves@gmail.com (L.M.N.); dafne.horovitz@iff.fiocruz.br (D.D.G.H.); guida.leticia@gmail.com (L.G.); leohfg@gmail.com (L.H.F.G.); danielapradocunha@gmail.com (D.P.C.); andrea.zin@iff.fiocruz.br (A.A.Z.); 3 Instituto de Genética Ocular, São Paulo 04552-050, Brazil; fabiana.louise@gmail.com; 4 Instituto Catarata Infantil, Rio de Janeiro 22250-040, Brazil

**Keywords:** genotype, phenotype, pediatric cataract, congenital cataract, microphthalmia, pediatric ophthalmology

## Abstract

Up to 25% of pediatric cataract cases are inherited, with half of the known mutant genes belonging to the crystallin family. Within these, crystallin beta B3 (*CRYBB3*) has the smallest number of reported variants. Clinical ophthalmological and genetic-dysmorphological evaluation were performed in three autosomal dominant family members with pediatric cataract and microphthalmia, as well as one unaffected family member. Peripheral blood was collected from all participating family members and next-generation sequencing was performed. Bioinformatics analysis revealed a novel missense variant c.467G>A/p.Gly156Glu in *CRYBB3* in all family members with childhood cataract. This variant is classified as likely pathogenic by ACMG, and no previous descriptions of it were found in ClinVar, HGMD or Cat-Map. The only other mutation previously described in the fifth exon of *CRYBB3* is a missense variant that causes a change in amino acid from the same 156th amino acid to arginine and has been associated with pediatric cataract and microphthalmia. To the best of our knowledge, this is the first time the c.467G>A/p.Gly156Glu variant is reported and the second time a mutation in CRYBB3 has been associated with microphthalmia.

## 1. Introduction

Cataract is defined as lens opacity that results in light scattering, reduced image clarity and visual acuity, as well as impaired contrast sensitivity [1,2]. In children, cataract may be congenital or acquired during childhood and can lead to irreversible visual impairment when it is not time-appropriately treated. Although it is a rare disease, with an overall prevalence ranging from 0.32 to 22.9 per 10,000 children (median 1.03) [2], it represents 21.3% of pediatric blindness worldwide, with higher frequencies in low- and middle-income countries [3]. Cataract in children must be diagnosed and managed early to avoid permanent vision impairment due to amblyopia.

Pediatric cataract can be isolated or associated with anterior chamber developmental anomalies, such as microcornea, microphthalmia and aniridia. They can also be related to multisystem genetic conditions, including chromosome disorders, developmental disorders or metabolic disorders [4]. Inherited congenital cataract comprises between 8.3 and 25% of cases [5], while causal genes have not been identified in one-third of the mapped loci [4]. About 37% of the known mutant genes in cataract families involve mutations in crystallin genes (1). There have been 11 mutations reported within the crystallin beta B3 *(CRYBB3*) [6]. Supplementary Table S1 describes previously reported mutations in *CRYBB3*, as well as phenotypes, family origins and inheritance patterns.

We aimed to determine the genetic cause of pediatric cataract related to microphthalmia in three members of a family with an autosomal dominant pattern of inheritance of pediatric cataract and microphthalmia phenotype using next-generation sequencing.

## 2. Materials and Methods

### 2.1. Study Design

A case report with clinical and next-generation sequencing of three members of the same autosomal dominant family (father and two children) with a history of pediatric cataract and microphthalmia, as well as one family member who did not present any ocular pathologies.

### 2.2. Clinical Evaluation

At evaluation, all three patients had already been submitted to cataract surgery, and detailed medical records were available for the two youngest. History of congenital TORCH infections (toxoplasmosis, rubella, cytomegalovirus, herpes simplex, syphilis, varicella zoster, zika), use of corticosteroids and ocular trauma were ruled out.

Ophthalmic exam with slit-lamp biomicroscopy, intraocular pressure (IOP), indirect ophthalmoscopy, ophthalmic ultrasonography and biometry for axial length measurement were performed in all individuals.

Genetic evaluation of family history and origins and pedigree, as well as existence of other systemic conditions, and clinic-morphological evaluation were performed by a clinical geneticist.

### 2.3. Genomic DNA Preparation

Peripheral blood samples were collected in EDTA tubes from the four family members. Genomic DNA was extracted from peripheral blood leucocytes using PureLink^®^ Genomic DNA Mini Kit Thermofisher (USA), according to the manufacturer’s protocol. DNA concentration in samples were determined by fluorometry using Invitrogen Qubit^®^ 4 Fluorometer. To assess DNA purity, a Spectrophotometer NanoDrop^®^ 2000 was used to evaluate ratio of the absorbance at 260/280 nm (average of 1.90 for all samples) and at 260/230 nm (average of 1.91 for all samples). DNA samples were stored at 4 °C prior to use.

### 2.4. Library Preparation and Clinical Exome Sequencing

DNA libraries from both parents and children were prepared with 50 ng of DNA using Clinical Exome Solution V2^®^ according to the manufacturer’s instructions (Sophia Genetics, Switzerland). This clinical exome solution covers 4493 genes related to the most common inherited diseases. Sequencing was performed using the NextSeq^®^ 500 system (Illumina, San Diego, CA, USA) using a multiplex system with 16 samples per run with the NextSeq^®^ kit. During library preparation, DNA fragments of 400 bp long on average were evaluated using the Bioanalyzer by Agilent.

### 2.5. Bioinformatics Analysis

Sequencing data were processed and analyzed using SOPHiA pipelines and DDM^®^ software (Switzerland), and the genetic variant calls were performed against the reference sequence of hg19 from the University of California Santa Cruz (UCSC) Genome Browser. The analysis strategy started with a virtual panel based on Human Phenotype Ontology (HPO) where 658 genes were found related to cataract in general (Supplementary Table S2). Genogram trio analysis was performed on affected and unaffected family members using the trio analysis tools available on SOPHiA DDM^®^.

During variant interpretation, we considered allele frequency using the Exome Aggregation Consortium database (ExAC), 1000 Genomes Project database, gnomAD and ABraOM, an online archive of Brazilian mutations [7]. Twelve predictors were considered for pathogenicity: BayesDel_addAF, DANN, DEOGEN2, EIGEN, FATHMM-MKL, LIST-S2, M-CAP, MVP, MutationAssessor, MutationTaster, SIFT and PrimateAI. The clinical significance of variants was evaluated with ClinVar, Polymorphism database (dbSNP) and Human Gene Mutation Database (HGMD). Cat-Map, an online chromosome map and reference database for cataract in humans and mice [6], was also searched for previous variant descriptions and clinical associations. Variant naming was based on reference sequence NM_004076.

### 2.6. Sanger Sequencing

In order to confirm the variant identified by clinical exome analysis, PCR amplification and bi-directional direct Sanger sequencing were performed using the oligonucleotide primers 5′CCTCCTTGACCTCTGTTCTGG3′ and 5′ GGCACTGATTCTGTTTGGAGC3′. Briefly, primers and PCR products were purified using PureLink^®^ (Invitrogen™) and sequenced on an automated sequencer (ABI 3730 Genetic analyzer, Applied Biosystems).

## 3. Results

**Figure 1:**
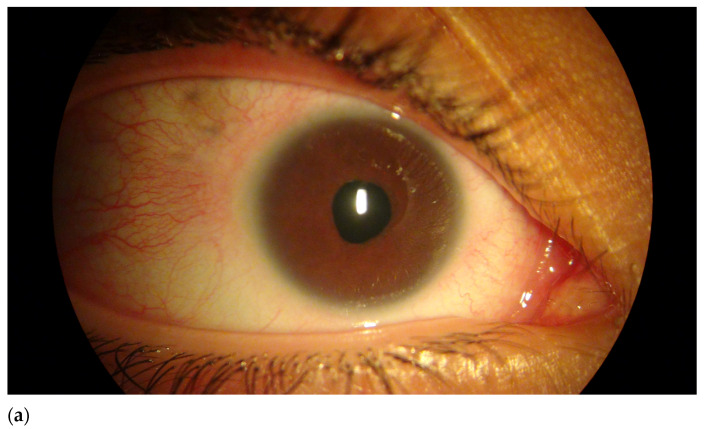

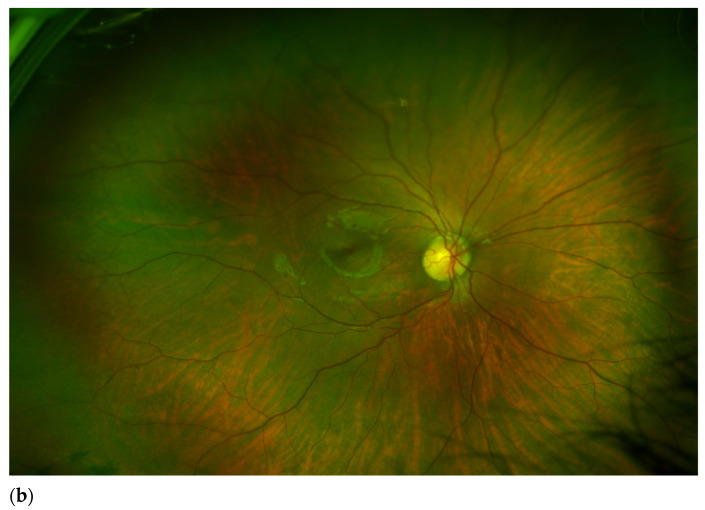
(**a**) Biomicroscopy photograph of proband’s microphthalmic eye. (**b**) Retinography showing no fundoscopic alterations in proband.

Proband was examined at 3 months old and had an unremarkable gestational and peripartum history. An ophthalmological exam revealed nystagmus and bilateral anterior polar cataract, as well as small corneas (7 mm diameter OU) and a reduced axial length (16.06 mm OD, 16.13 mm OS) for her age. Ultrasonography showed no alterations other than lens opacification and a reduced axial length. A diagnosis of bilateral microphthalmia and congenital cataracts was made and lensectomy with anterior vitrectomy was performed on both eyes. Aphakic glaucoma developed four years after surgery on both eyes and was managed with topical antiglaucoma drugs. Figure 1 shows (a) small corneas compatible with the microphthalmia phenotype, as well as an (b) unremarkable fundoscopy.

Family history revealed that the proband’s father was diagnosed with bilateral cataracts at three years old, as well as microphthalmia. The eldest brother also presented with nystagmus and microphthalmia and was diagnosed with cataracts at 18 months old. Two years after lensectomy, the brother presented pain in the left eye. Ophthalmological examination revealed aphakia with a shallow anterior chamber and high intraocular pressure. A diagnosis of aqueous misdirection was made and a surgical approach indicated. Since both the father and eldest son were not initially operated on at our facilities, the records of their cataract phenotype prior to surgery were not available. At examination, the father was microphthalmic, with nystagmus and aphakic bilaterally. Table 1 summarizes the clinical findings of the three family members with cataract.

**Figure 2:**
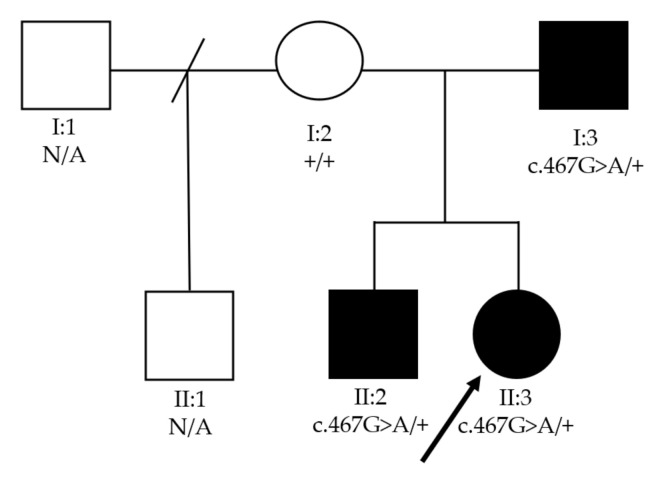
Family pedigree of *CRYBB3* patients with two generations depicted. Black circles and squares indicate individuals affected by pediatric cataract and microphthalmia. N/A—data not available.

**Figure 3:**
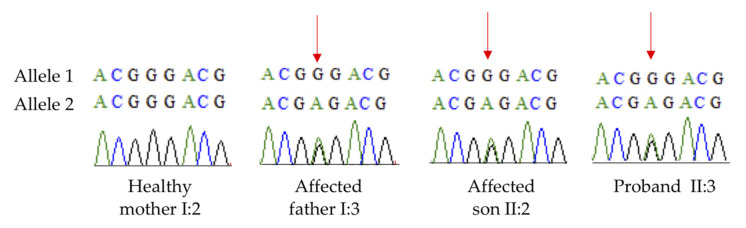
Confirmation of variant by Sanger sequencing of affected and unaffected family members.

Next-generation sequencing revealed a novel missense variant c.467G>A/p.Gly156Glu in *CRYBB3* in all family members with childhood cataract. No previous descriptions of it were found in ClinVar, HGMD or Cat-Map. Not only is p.Gly156 highly conserved among several species, the variant c.467G>A/p.Gly156Glu in *CRYBB3* segregates accordingly in family members (Figure 2). The variant in the same codon c.466G>A (p.Gly156Arg) of *CRYBB3*, a consolidated gene associated with pediatric cataract, has been described in other ethnic groups, such as Chinese [8] and Turkish families [9]. In addition, c.467G>A/p.Gly156Glu is located in a well-established functional domain (beta/gamma crystallin ‘Greek key’4′) that is also absent from consulted controls. The variant’s pathogenicity is also supported by 11 of the 12 predictors, except PrimateAI. Considering the aforementioned, the variant c.467G>A/p.Gly156Glu in *CRYBB3* is classified as likely pathogenic by ACMG criteria [10]. Figure 3 shows confirmation of the variant performed by Sanger sequencing, which was found in all affected family members and not in the healthy parent.

## 4. Discussion

To the best of our knowledge, this is the second time a mutation in *CRYBB3* has been associated with microphthalmia. It is a novel, likely pathogenic variant presenting with an autosomal dominant pattern of inheritance.

Pediatric cataract is a phenotypically and genetically heterogeneous disorder and inherited traits showed interfamily and intrafamily variability [11]. To date, there are 39 cytogenetic loci known to be involved in pediatric cataract. About half of the known mutant genes in families with cataract involve mutations in crystallin genes [6].

Crystallins are the most abundant soluble proteins in the ocular lens (80–90%) and account for its optical transparency and high refractive index [12,13]. They are subdivided into alfa (40%), beta (35%) and gamma (25%) according to the order of their elution on gel exclusion chromatography [1,12,14]. The present study reports a novel mutation which disrupts the beta-crystallin Greek key functional domain, as is the case in most of the congenital cataract’s pathogenic missense mutations in this gene [15].

beta-crystallins can also be subdivided into four acidic (A1, A2, A3, A4) and three basic (B1, B2, B3) isoforms, depending on the isoelectric point and the terminal extension [16]. The least well studied is the crystallin beta B3 (*CRYBB3*) [17]. There are 9 mutations reported in *CRYBB3*, 23 in *CRYBB1* and 35 in *CRYBB2* [6].

Seventy percent of pediatric cataract are isolated [1], and, as expected, most of the *CRYBB3* mutations reported in the Cat-Map are also isolated [6]. However, pediatric cataract is also associated with other ocular malformations in 15% of cases, including microphthalmia, aniridia, other anterior chamber developmental anomalies or retinal degenerations [1]. Another report of that same *CRYBB3* missense variant c.466G>A (p.Gly156Arg) has included microphthalmia as an additional phenotype of congenital cataract [9].

The phenotype of all these c.466G>A (p.Gly156Arg) mutations was reported as bilateral nuclear cataract [8,9,18]. Conversely, in our present study, the novel c.467G>A (p.Gly156Glu) variant clinically manifested as bilateral anterior polar cataract with microphthalmia.

Interestingly, glaucoma also developed after surgery in the patient with microphthalmia and congenital cataract described by Sekeroglu et al. [9], as was seen in our proband. Glaucoma is a possible event after lensectomy in pediatric patients, its incidence varying between 10 and 58% [19]. According to Chak et al. [20], the age at which surgery is performed is the only factor associated with the development of glaucoma—the earlier surgery is performed, the higher the risk is. An association with microphthalmia and the development of glaucoma was not found. Similar mutations leading to the same complications raise the question as to whether the genotype of patients could be associated with the development of this complication. Are there genes only associated with alterations in the crystalline or could there be other anterior segment malformations associated with and responsible for the development of glaucoma? Further studies are necessary in order to evaluate such a hypothesis.

Genetic diagnosis of developmental eye disorders remains a challenge worldwide due to diverse phenotypes and genetic heterogeneity [18]. However, the evolving knowledge of genetics in medicine enables the phenotypic–genotypic correlation and a better understanding of the disease’s physiopathology. Identification of the causative mutations allows genetic counseling. The benefit regarding de novo mutations is even greater, since the pattern of inheritance and risk for other family members is impossible to predict from the pedigree.

## 5. Conclusions

Timely pediatric cataract surgical treatment is efficient and does not depend on molecular diagnosis. Nonetheless, the latter is important since it may provide knowledge toward the development of new therapeutic possibilities. Once the causative mechanism of the cataract is identified, the degenerative progressive damage could be delayed and surgical treatment avoided.

Therefore, we believe that our finding can contribute to better understand the role of the CRYBB3 protein in inherited childhood cataract associated with microphthalmia.

## Figures and Tables

**Table 1:** Clinical features of 3 family members with cataract.

Patient	Gender	Age at Time of Diagnosis (months)	Bilateral Cataract Phenotype	Other Ocular Abnormalities
I:3	M	36	Unknown	Microphthalmia
II:2	M	18	Unknown	Microphthalmia, aqueous misdirection
II:3	F	3	Anterior polar	Microphthalmia, aphakic glaucoma

I:3—father; II:2—son; II:3—daughter; M—male; F—female.

## Data Availability

Data is available on the public online database CAT-MAP and from related publications and supplements. This data can be found here: https://cat-map.wustl.edu/.

## Supplementary Materials

The following are available online at https://www.mdpi.com/article/10.3390/genes12071069/s1, Table S1: Previously reported mutations in *CRYBB3*. Table S2: List of 658 genes related to cataract available at Human Phenotype Ontology.
