# Supplementary material for: Novel Mutation in CRYBB3 Causing Pediatric Cataract and Microphthalmia
Source: Genes (Basel). 2021 Jul 13;12(7):1069. doi: 10.3390/genes12071069 (PMC8308043; doi:10.3390/genes12071069)
Supplement: Supplementary file 1 [file genes-12-01069-s001.zip › genes-1253197-supplementary.pdf]

Supplementary Materials

Table S1. Previously reported mutations in *CRYBB3*.

Nucleotide Change	Amino Acid Change	Type of Mutation	Inheritance Pattern	Family Origin	Cataract Phenotype	Other Phenotype	Reference
c.75+1 G>A		Splice site	AD	China	Zonular	N/A	Yu (2021), Mol Genet Genomic Med 9:1617.
c.224G>A	p.Arg75His	Missense	AD	Denmark	N/A	Microcornea	Hansen (2009), Investig Ophthalmol Vis Sci, 50:3291.
c.226G>A	p.Gly76Arg	Missense	Sporadic	China	Cortical	N/A	Dan Li (2016), Mol Vis, 22:589.
c.314G>A	p.Arg105Gln	Missense	Sporadic	China	Total	N/A	Dan Li (2016), Mol Vis, 22:589.
c.466G>A	p.Gly156Arg	Missense	Sporadic	China	Nuclear	N/A	Dan Li (2016), Mol Vis, 22:589.
c.466G>A	p.Gly156Arg	Missense	AD	Pakistan	Nuclear	N/A	Jackson (2020), Am J Med Genet C Semin Med Genet 184:578.
c.466G>A	p.Gly156Arg	Missense	AD	Turkey	Nuclear	Microphthalmia, aphakic glaucoma	Sekeroglu (2020), Mol Syndromol 11:302.
c.493G>C	p.Gly165Cys	Missense	AR	Pakistan	Nuclear	N/A	Riazuddin et al., 2005.

c.493G>C	p.Gly165Cys	Missense	AR	Australia	N/A	N/A	Ma (2016), Human Mutat 37:371.
c.581T>A	p.Val194Glu	Missense	AD	Italy	Posterior polar, nuclear, anterior polar plus cortical	Glaucoma, incomplete penetrance	Reis (2013), Hum Genet 132:761.
c.634T>C	p.*212Argext*40	Missense	AD	Australia	N/A	N/A	Ma (2016), Human Mutat 37: 371.

(AD – autosomal dominant; AR – autosomal recessive; N/A – non applicable).

Supplementary Material

Table S2. List of 658 genes related to cataract available at Human Phenotype Ontology.

ABCA12
ABCA2
ABCA4
ABHD12
ABHD5
ACTB
ADA2
ADAMTS10
ADAMTS18
ADAMTSL4
ADGRV1
ADNP
AFF4
AGA
AGBL5

AGK
AHI1
AHR
AIPL1
AIRE
AKT1
ALDH18A1
ALDH6A1
ALDOB
ALG2
ALG8
ALMS1
ALX1
ALX3
AMER1
AMMECR1
ANAPC1
ANO10
AP1S1
APC
APC2
ARHGAP31
ARHGEF18
ARID1A
ARID1B
ARID2
ARL2BP
ARL3

ARL6
ARSG
ARSL
ATAD3A
ATOH7
ATP6V1A
ATP6V1B2
ATP8A2
B3GALNT2
B3GLCT
B4GALNT1
B4GAT1
B9D1
B9D2
BAP1
BBIP1
BBS1
BBS2
BCOR
BCS1L
BEST1
BFSP1
BFSP2
BMP4
BRCA1
BRCA2
BRF1
BRIP1

BTNL2
BUB1
BUB1B
C4A
C8orf37
CA4
CA8
CASK
CAV1
CBS
CC2D2A
CCDC28B
CDH11
CDH23
CDHR1
CENPF
CEP164
CEP290
CEP55
CEP57
CEP78
CERKL
CFAP410
CHMP4B
CHRDL1
CIB2
CLN3
CLPB

CLRN1
CNBP
CNGA1
CNGB1
CNGB3
COG4
COL11A1
COL18A1
COL2A1
COL4A1
COL4A3
COL4A4
COL4A5
COL7A1
COL9A1
COL9A2
COL9A3
COMT
COX1
COX2
COX3
COX7B
CPAMD8
CPT2
CRB1
CREBBP
CRPPA
CRX

CRYAA
CRYAB
CRYBA1
CRYBA2
CRYBA4
CRYBB1
CRYBB2
CRYBB3
CRYGB
CRYGC
CRYGD
CRYGS
CSPP1
CTC1
CTDP1
CYP1B1
CYP27A1
CYP7B1
CYTB
DACT1
DAG1
DDB2
DGUOK
DHCR7
DHDDS
DHX38
DKC1
DLL4

DMPK
DNM1L
DNMT1
DOCK6
DPAGT1
DPF2
DSG4
DYNC2H1
DYNC2I1
DYNC2I2
EBP
EED
ELN
ELP4
ENTPD1
EOGT
EP300
EPG5
EPHA2
ERCC1
ERCC2
ERCC3
ERCC4
ERCC5
ERCC6
ERCC8
ESCO2
ESPN

ETFA
ETFB
ETFDH
EYS
FAM111A
FAM111B
FAM126A
FAM161A
FANCA
FANCB
FANCC
FANCD2
FANCE
FANCF
FANCG
FANCI
FANCL
FANCM
FAR1
FAS
FBN1
FBXL4
FGF3
FGF5
FH
FIBP
FIG4
FKRP

FKTN
FLI1
FLNA
FLNB
FLVCR1
FOXC1
FOXC2
FOXE3
FSCN2
FTL
FYCO1
FZD4
GABRD
GALK1
GALM
GALT
GATA1
GBA2
GCNT2
GDF6
GDNF
GEMIN4
GFER
GJA1
GJA3
GJA8
GJB3
GJB4

GJB6
GLA
GMPPB
GNA11
GNAQ
GNAS
GNPAT
GP1BB
GSN
GSR
GTF2E2
GTF2H5
GTPBP2
GUCA1B
GUCY2D
HARS1
HBB
HCCS
HDAC8
HGSNAT
HLA-A
HLA-B
HLA-DRB1
HMX1
HNRNPA1
HNRNPA2B1
HNRNPDL
HSF4

HSPG2
HTRA2
IARS2
IDH3B
IFT140
IFT172
IFT80
IKBKG
IL10
IL23R
IMPDH1
IMPG2
INPP5E
INPP5K
INTS1
INVS
IQCB1
ITM2B
ITPA
JAG1
JAM3
KANSL1
KCNA4
KCNH1
KCNJ13
KCNN3
KCTD1
KDSR

KIAA1109
KIAA1549
KIF11
KIF1B
KIZ
KLHL7
KLLN
KMT2A
KRT25
KRT71
KRT74
KRT81
KRT83
KRT86
LAMB1
LARGE1
LCA5
LEMD2
LIG4
LIM2
LIPH
LMNA
LMX1B
LONP1
LOXL1
LPAR6
LRAT
LRP2

LRP4
LRP5
LSS
LTBP2
MAB21L2
MAD2L2
MAF
MAFA
MAK
MAN2B1
MAP2K2
MAX
MED25
MEFV
MERTK
MFRP
MIP
MIPEP
MIR140
MIR184
MIR204
MKS1
MMP1
MORC2
MPLKIP
MSMO1
MTAP
MVK

MYH9
MYMK
MYO7A
MYSM1
NAA10
NACC1
NAGA
ND1
ND5
ND6
NDP
NDUFB11
NEK2
NEK9
NEU1
NF2
NHP2
NHS
NIPBL
NMNAT1
NOD2
NOP10
NOTCH1
NOTCH2
NPHP1
NPHP3
NPHP4
NPM1

NR2E3
NRL
NSD1
NSUN2
NUP188
OAT
OCLN
OCRL
OFD1
OPA1
OPA3
OTX2
P3H2
PAH
PALB2
PARN
PAX2
PAX6
PCARE
PCDH15
PCYT1A
PDE6A
PDE6B
PDE6G
PDZD7
PEX1
PEX10
PEX11B

PEX12
PEX13
PEX14
PEX16
PEX19
PEX2
PEX26
PEX3
PEX5
PEX6
PEX7
PHF6
PHGDH
PHYH
PIGY
PIK3C2A
PIK3CA
PIK3R1
PITX2
PITX3
PLCG2
PLK4
PLOD3
PMM2
PNPLA6
PNPT1
POLG
POLG2

POLR1C
POLR1D
POLR3A
POMGNT1
POMGNT2
POMK
POMT1
POMT2
PQBP1
PRCD
PRDM16
PROM1
PRPF3
PRPF31
PRPF4
PRPF6
PRPF8
PRPH2
PRUNE1
PSAT1
PTCH1
PTCH2
PTEN
PTH
PTH1R
PTPN22
PXDN
RAB18

RAB3GAP1
RAB3GAP2
RAD21
RAD51
RAD51C
RALGAPA1
RB1
RBP3
RBPJ
RD3
RDH11
RDH12
RECQL4
REEP6
RERE
RET
RFWD3
RGR
RHO
RHOA
RIC1
RLBP1
RNF113A
RNF13
ROM1
RP1
RP2
RP9

RPE65
RPGR
RPGRIP1
RPGRIP1L
RPL11
RPL15
RPL18
RPL26
RPL27
RPL35
RPL35A
RPL5
RPS10
RPS15A
RPS17
RPS19
RPS24
RPS26
RPS27
RPS28
RPS29
RPS6KA3
RPS7
RRM2B
RS1
RSPO2
RTEL1
RXYLT1

SAG
SALL1
SALL2
SALL4
SC5D
SCAPER
SDCCAG8
SDHB
SDHC
SDHD
SEC23A
SEC23B
SEC31A
SEMA4A
SETD2
SETD5
SF3B1
SHROOM4
SIL1
SIPA1L3
SIX6
SKI
SLC16A12
SLC25A13
SLC25A4
SLC2A1
SLC33A1
SLC40A1

SLC4A4
SLC7A14
SLX4
SMAD3
SMAD4
SMARCA4
SMARCB1
SMARCC2
SMARCD1
SMARCE1
SMC1A
SMC3
SMCHD1
SNRNP200
SOX11
SOX4
SPATA7
SPRTN
SRD5A3
STAT4
STX16
SUFU
SUMF1
TARS1
TBC1D20
TBC1D24
TBR1
TBX1

TCOF1
TCTN2
TDRD7
TELO2
TERC
TERT
TFAP2A
TGFBI
TGM3
TINF2
TKFC
TKT
TMEM107
TMEM127
TMEM216
TMEM231
TMEM67
TMEM70
TMTC3
TONSL
TOPORS]
TRAF3IP1
TRAPPC11
TRIM44
TRIP13
TRNC
TRNF
TRNK

TRNL1
TRNQ
TRNS1
TRNS2
TRNV
TRNW
TRPV4
TSR2
TTC8
TUB
TUBB2B
TUBG1
TUBGCP6
TULP1
TWNK
UBA5
UBE2A
UBE2T
UNC45B
USB1
USH1C
USH1G
USH2A
USP45
USP8
USP9X
VAC14
VCAN

VCP
VHL
VIM
VLDLR
VSX2
WDPCP
WDR19
WDR35
WDR73
WDR81
WFS1
WHRN
WNT3
WRAP53
WRN
WT1
XPA
XPC
XRCC2
XRCC4
XYLT2
YAP1
ZBTB20
ZEB2
ZNF335
ZNF408
ZNF513
